# A case report of prenatal diagnosis of fetal alloimmune thrombocytopenia: A CARE-compliant article

**DOI:** 10.1097/MD.0000000000026092

**Published:** 2021-06-18

**Authors:** Jing Fu, Ruojin Yao, Wenjing Yong

**Affiliations:** a Reproductive Medicine Center; b Department of Obstetrics, Xiangya Hospital, Central South University, Changsha, Hunan, China.

**Keywords:** fetal alloimmune thrombocytopenia, fetal intracranial hemorrhage, fetal thrombocytopenia, prenatal diagnosis, umbilical cord puncture

## Abstract

**Rationale:**

Fetal alloimmune thrombocytopenia (FAIT) is a serious life-threatening disease caused by platelet-antigen incompatibility between the mother and fetus. FAIT can lead to fetal thrombocytopenia, intracranial hemorrhage (ICH), fetal death and severe neurological disorders after birth. Noninvasive prenatal diagnosis technology has not been widely used in China, and thus few cases of FAIT can be diagnosed prenatally. In this study, we report a case of prenatal diagnosis and treatment of FAIT.

**Patient concerns:**

A 29-year-old female was admitted at 32 weeks’ gestational age (GA). Fetal ultrasound at 32 weeks’ GA showed a hemorrhagic focus area in the left lateral ventricle and the sign of severe fetal anemia. Hence, fetal umbilical cord puncture was ordered to identify the etiology.

**Diagnoses:**

The fetal cord blood test revealed a normal hemoglobin level but severe fetal thrombocytopenia (platelet count, 23 × 10^9^/L). Antibodies of human platelet antigens and human leukocyte antigens between mother and fetus were positive, and thus the diagnosis of FAIT was confirmed.

**Interventions:**

The patient refused intravenous immunoglobulin (IVIG) therapy owing to financial consideration. She was treated with dexamethasone acetate tablets (Xianju Company, China) 0.75 mg twice a day until delivery and cesarean section was performed at 34 weeks’ GA. The newborn received postnatal anti-platelet antibody treatment.

**Outcomes:**

The platelet count of the newborn progressively decreased until the third day after birth and it increased to normal level after postnatal treatment. The neonatal cerebral ultrasound showed the area of hemorrhage was in the process of absorption. During the postnatal one-year follow-up, the neonate showed normal developmental milestones and had no abnormal signs of neurological symptoms.

**Lessons:**

For FAIT, the fetal umbilical cord puncture can be carried out by skilled fetal medical teams. Dexamethasone acetate tablets can be an alternative choice for patients from underdeveloped areas.

## 1 Introduction

Fetal alloimmune thrombocytopenia (FAIT) is a life-threatening disease with a global incidence of 1/2000 to 1/3000.^[1,2]^ It occurs when the maternal immunoglobulin G antibodies activated by human platelet antigens (HPAs) act on fetal platelets, resulting in fetal thrombocytopenia.^[3]^ It is the most common cause of fetal intracranial hemorrhage (ICH) and can often lead to fetal death or severe neurological disorders after birth. The clinical manifestations of FAIT are different, including asymptomatic thrombocytopenia, skin hemorrhage, severe organ hemorrhage, ICH, stillbirth, neonatal asphyxia and perinatal infection.^[4,5]^

Currently, the diagnosis of FAIT is mostly made postnatally because the noninvasive prenatal diagnosis technology has not been widely used in China, and thus few cases of FAIT can be diagnosed prenatally. In this study, we report a case of prenatal diagnosis and treatment of FAIT. Informed consent was obtained from the patient for publication of this case.

## 2 Case report

The patient was a 29-year-old G1P0 Chinese female with the AB, Rh positive blood type. She had no significant medical history and symptoms. At 22 weeks’ gestational age (GA), the fetal ultrasound showed that the echo of the intestinal canal in the right lower abdomen of the fetal was slightly enhanced, and no obvious abnormality was found in amniocentesis result. At 23+2 weeks’ GA, the fetal ultrasound indicated fetal growth restriction (FGR). Thus, symptomatic support treatment, such as fluid replacement and improvement of placental microcirculation was applied during the next week. The fetal ultrasound at 32 weeks’ GA showed FGR still exist, while there was a 9 × 7 × 9 mm mixed echo area in fetal brain at the junction of the anterior horn and the body of the left lateral ventricle, which might be a hemorrhagic focus. However, the fetal middle cerebral artery peak systolic velocity (MCA-PSV) increased to 1.69 MoM. Due to the MCA-PSV value was higher than 1.5 MoM, which is a recognized predictor of severe fetal anemia,^[6,7]^ we decided to carry out a fetal umbilical cord puncture in order to identify the etiology. The blood type of the fetus was AB, Rh positive. Both the direct antiglobulin test and irregular blood group antibody screening were negative. Since the hemoglobin level of fetal cord blood test was normal and the hemolysis test was negative, fetal anemia was excluded. However, the fetal cord blood test revealed a severe fetal thrombocytopenia (platelet count, 23 × 10^9^/L). Antibodies of HPAs and human leukocyte antigens between mother and fetus were positive, and thus the diagnosis of FAIT was confirmed. The intravenous immunoglobulin (IVIG) therapy was first recommended but the patient refused owing to financial consideration. Then she was treated with dexamethasone acetate tablets (Xianju Company, China) 0.75 mg twice a day until delivery. At 33+3 weeks’ GA, the fetal ultrasound showed that the hemorrhagic focus area increased to 13 x 9 x 12 mm and the MCA-PSV increased to 1.90 MoM. Multi-disciplinary team advised that with the increase of gestational weeks, fetal platelets would be continuous destructed, which might lead to progressive thrombocytopenia and increase the risk of fetal ICH. So, cesarean section was performed at 34+2 weeks’ GA. There were no bleeding spots or ecchymosis on the newborn's skin. The Apgar scores of the newborn was 9 at 1 minute (skin color deducted 1 point), 10 at 5 minutes and 10 at 10 minutes, respectively. The platelet count of the newborn was 70 × 10^9^ /L, and progressively decreased to 12 × 10^9^ /L until the third day after birth. The neonatal cerebral ultrasound on day 5 and day 12 showed that the area of hemorrhage was in the process of absorption. The platelet count of the newborn increased gradually to 59 × 10^9^ /L after one-month postnatal anti-platelet antibody treatment including methylprednisolone sodium succinate (Pfizer Manufacturing Belgium NV) 1.6 mg/kg for 3 days, IVIG (Hualan Biological Engineering Inc, China) 1 g/kg twice and prednisone acetate tablets (Xianju Company, China) 2 mg/kg every 12 hours (gradually reduced to 0.75 mg per day till 1 month). During the postnatal one-year follow-up, the neonate showed normal developmental milestones and had no abnormal signs of neurological symptoms.

## 3 Discussion

At present, the recommendation of prenatal diagnosis procedures for FAIT are as follows: first, fetal HPAs typing is detected in maternal, paternal serum and amniotic fluid to analyze whether there is HPAs incompatibility; then it is recommended to use monoclonal antibody immobilization of platelet specific antigen assay (MAIPA) to analyze and identify maternal platelet specific alloantibodies, or to detect maternal serum autoantibodies.^[8–10]^ Limited by the advanced skills and costs, non-invasive prenatal diagnosis technology has not been universally available in China and there is few cases of FAIT can be successfully diagnosed prenatally and received intervention.

In this case, ultrasound demonstrated FGR at 23+2 weeks’ GA, and the MCA-PSV of the fetus was increased higher than 1.5 Mom at 32 weeks’ GA. Thus, we considered a severe anemia may occurred. Although umbilical cord puncture is not routinely recommended for fetal ICH, the progressive deterioration of fetal anemia could lead to fetal death.^[11]^ Therefore, umbilical cord puncture was performed after comprehensive consideration. The hemoglobin level of fetal cord blood test was normal, suggesting the fetal anemia could be excluded. However, the platelet count of the fetus was only 23 × 10^9^/L, presenting a severe thrombocytopenia. The symptoms of thrombocytopenia and ICH in the fetus and the presence of anti-platelet antibodies in both the mother and fetus can lead to the diagnosis of FAIT. It must be stated that, in order to reduce complications such as progressive thrombocytopenia and fetal heart rate decrease caused by thrombocytopenia, the umbilical cord puncture should be carried out in medical centers with skilled fetal medical teams when necessary.

It is generally believed that thrombocytopenia caused by maternal antiplatelet antibodies is the key factor in the occurrence of ICH in FAIT.^[12]^ But some studies illustrate that impaired angiogenesis may also play an important role in FAIT related ICH instead of thrombocytopenia. Issaka proved that the angiogenic signaling could be disturbed by β3 integrin in the murine models of FAIT. Besides, they also thought the endothelial cell apoptosis and decreased vessel density that were induced by β3 integrin played critical role in the occurrence of ICH in FAIT.^[13–15]^ Thus under the circumstance of ICH, invasive diagnostic approaches should be considered when it is carried out in medical centers with skilled fetal medical teams.

Furthermore, β3 integrin may be related to FGR. A Norwegian study indicated that when the levels of maternal HPA 1a were higher, the birthweight of boys were lower.^[16]^ They investigated anti-HPA 1a antibodies might reduce placenta function through damaging vascular and inhibiting angiogenesis. Besides, Eksteen found anti-HPA 1a antibodies could disturb trophoblast functions and effect the placental development in vitro model.^[15]^ In this case, fetal anemia was excluded after umbilical cord puncture, and fetal genetic material abnormality was excluded by amniocentesis. Therefore, it is considered that the existence of FGR may be related to FAIT.

For the prenatal intervention of FAIT, the first-line treatment recommended is IVIG, and the second-line treatment is glucocorticoid.^[11,17,18]^ This case was treated with dexamethasone acetate tablets. Compared with other glucocorticoids, dexamethasone can not only reduce platelet antibodies in fetus through the placental barrier, but also promote the development and maturity of fetal lungs. Besides, it is more economical in comparison with IVIG. For patients from underdeveloped areas, like this case, dexamethasone acetate tablets may be an alternative choice.

## 4 Conclusion

For FAIT, the fetal umbilical cord puncture can be carried out by skilled fetal medical teams. Dexamethasone acetate tablets can be an alternative choice for patients from underdeveloped areas.

## Author contributions

**Resources:** Ruojin Yao.

**Writing – original draft:** Jing Fu.

**Writing – review & editing:** Wenjing Yong.
